# Radiotherapy fractionation for the palliation of uncomplicated painful bone metastases – an evidence-based practice guideline

**DOI:** 10.1186/1471-2407-4-71

**Published:** 2004-10-04

**Authors:** Jackson Sai-Yiu Wu, Rebecca KS Wong, Nancy S Lloyd, Mary Johnston, Andrea Bezjak, Timothy Whelan, the Supportive Care Guidelines Group of Cancer Care Ontario

**Affiliations:** 1 Department of Radiation Oncology, Tom Baker Cancer Centre, Calgary, Alberta, Canada; 2 Department of Radiation Oncology and the Princess Margaret Hospital, University of Toronto, Toronto, Ontario, Canada; 3 Department of Clinical Epidemiology & Biostatistics, McMaster University, Hamilton, Ontario, Canada; 4 Division of Radiation Oncology, Juravinski Cancer Centre and the Department of Medicine, McMaster University, Hamilton, Ontario, Canada

## Abstract

**Background:**

This practice guideline was developed to provide recommendations to clinicians in Ontario on the preferred standard radiotherapy fractionation schedule for the treatment of painful bone metastases.

**Methods:**

A systematic review and meta-analysis was performed and published elsewhere. The Supportive Care Guidelines Group, a multidisciplinary guideline development panel, formulated clinical recommendations based on their interpretation of the evidence. In addition to evidence from clinical trials, the panel also considered patient convenience and ease of administration of palliative radiotherapy. External review of the draft report by Ontario practitioners was obtained through a mailed survey, and final approval was obtained from the Practice Guidelines Coordinating Committee.

**Results:**

Meta-analysis did not detect a significant difference in complete or overall pain relief between single treatment and multifraction palliative radiotherapy for bone metastases. Fifty-nine Ontario practitioners responded to the mailed survey (return rate 62%). Forty-two percent also returned written comments. Eighty-three percent of respondents agreed with the interpretation of the evidence and 75% agreed that the report should be approved as a practice guideline. Minor revisions were made based on feedback from the external reviewers and the Practice Guidelines Coordinating Committee. The Practice Guidelines Coordinating Committee approved the final practice guideline report.

**Conclusion:**

For adult patients with single or multiple radiographically confirmed bone metastases of any histology corresponding to painful areas in previously non-irradiated areas without pathologic fractures or spinal cord/cauda equine compression, we conclude that:

• **Where the treatment objective is pain relief, a single 8 Gy treatment, prescribed to the appropriate target volume, is recommended as the standard dose-fractionation schedule for the treatment of symptomatic and uncomplicated bone metastases.**

Several factors frequently considered in clinical practice when applying this evidence such as the effect of primary histology, anatomical site of treatment, risk of pathological fracture, soft tissue disease and cord compression, use of antiemetics, and the role of retreatment are discussed as qualifying statements.

Our systematic review and meta-analysis provided high quality evidence for the key recommendation in this clinical practice guideline. Qualifying statements addressing factors that should be considered when applying this recommendation in clinical practice facilitate its clinical application. The rigorous development and approval process result in a final document that is strongly endorsed by practitioners as a practice guideline.

## Background

Radiotherapy is a well-recognized, effective modality in the palliative treatment of painful bone metastases. Bone metastases are a common manifestation of distant relapse from many types of malignant tumours, especially from cancers of the lung, breast, and prostate. With the advent of effective systemic therapies and improvements in supportive care, cancer patients are expected to live longer and may suffer from metastatic disease for a considerable length of time. Many patients with bone metastases suffer from compromised mobility and performance status.

The optimal dose-fractionation schedule for the treatment of bone metastases is unclear. Two surveys of Canadian patterns of practice found that various fractionation schedules are employed by radiation oncologists, ranging from a single large-dose fraction (e.g., 8 Gy) to a more prolonged course of 30 Gy/10 fractions over 2 weeks [1,2]. It has been suggested that the choice of fractionation is influenced not only by patient-related factors but also by physician education and attitudes, treatment toxicity, resource utilization, and departmental policy [3-7]. While different clinicians may associate "*optimal*" with different treatment goals, one could recommend that a "*preferred*" dose-fractionation is one that provides pain relief without undue toxicity and is least onerous to the patient.

During the past decade, significant clinical trial efforts have been devoted to comparing single large-dose radiation (8 Gy to 10 Gy) with multifraction regimens (five to ten fractions) [8-14]. The two largest trials were published in 1999 by the Bone Pain Trial Working Party [10] and the Dutch Bone Metastasis Study group [11]. Results of a Canadian study were presented at the Canadian Association of Radiation Oncologists (CARO) meeting in 2000 and reported in an abstract for the 2000 meeting of the American Society for Therapeutic Radiology and Oncology (ASTRO) [9]. Those randomized trials should provide substantial evidence to address the question of a "preferred" fractionation for the majority of patients with bone metastases.

This provincial guideline was initiated to summarize the evidence and to provide recommendations on the preferred standard radiotherapy fractionation schedule for the treatment of painful bone metastases.

## Clinical practice guideline development

This practice guideline was developed by Cancer Care Ontario's Practice Guidelines Initiative (PGI), using the methods of the Practice Guidelines Development Cycle [15]. The practice guideline report is a convenient and up-to-date source of the best available evidence on the preferred dose-fractionation of radiotherapy for the treatment of uncomplicated painful bone metastases, developed through systematic reviews, evidence synthesis, and input from practitioners in Ontario. The report is intended to promote evidence-based practice. The PGI is editorially independent of Cancer Care Ontario and the Ontario Ministry of Health and Long-term Care. The PGI has a formal standardized process to ensure the currency of each guideline report. This process consists of the periodic review and evaluation of the scientific literature and, where appropriate, integration of this literature with the original guideline information.

Evidence was selected and summarized by four members of the Supportive Care Guidelines Group (SCGG) and methodologists. Members of the SCGG disclosed potential conflict of interest information, reviewed the analysis of the evidence, and prepared draft recommendations. The SCGG includes palliative care physicians, medical and radiation oncologists, psychiatrists, nurses, psychologists, a chaplain, an anesthetist, a surgeon, methodologists, and administrators. After reviewing the evidence and considering issues of patient convenience and resource utilization, the SCGG reached consensus on draft recommendations. The systematic review and meta-analysis, conducted as the initial step in formulating this practice guideline, has been described elsewhere [16].

External review by Ontario practitioners was obtained through a mailed survey consisting of items that address the quality of the draft practice guideline report and recommendations and whether the recommendations should serve as a practice guideline. The efficacy of the practitioner feedback survey process has been previously described [17]. Final approval of the original guideline report was obtained from the Practice Guidelines Coordinating Committee (PGCC).

## Methods

### External review – Ontario practitioner feedback

Practitioner feedback was obtained through a mailed survey of 95 radiation oncologists across Ontario. The survey consisted of items evaluating the methods, results, and interpretive summary used to inform the draft recommendation and whether the draft recommendation should be approved as a practice guideline. Written comments were invited. Follow-up reminders were sent at two weeks (post card) and four weeks (complete package mailed again). The SCGG reviewed the results of the survey.

### Results of practitioner feedback

Fifty-nine of the 95 surveys were returned (62% return rate). Key results of the practitioner feedback survey are summarized in Table 1. The survey results indicated that 83% of respondents agreed with the interpretation of the evidence and 74% agreed with the draft recommendations as stated. Seventy-five percent of respondents agreed that the report should be approved as a practice guideline. Twenty-one respondents (42%) also provided written comments. The final recommendation was revised to reflect feedback from practitioners and currently applies to patients for whom the treatment objective is pain relief.

### Practice guidelines coordinating committee approval process

The practice guideline report was circulated to PGCC members for review and approval. Eleven of the fourteen PGCC members completed and returned ballots. Ten PGCC members approved the practice guideline report as written, and one member approved the guideline and provided suggestions for consideration by the SCGG. Suggestions made were to reword the Target Population section and to clarify the second qualifying statement. The SCGG agreed with the suggestions and modified the guideline accordingly.

## Discussion

The preferred radiotherapy dose-fractionation schedule for the palliation of uncomplicated painful bone metastases has been a controversial subject [18-21]. The goal of our systematic review was to enable guideline developers and practitioners to determine whether the available evidence supports the notion of a "standard" dose-fractionation. "*Standard*" refers to what is applicable to the majority of patients, with a preference for patient convenience and ease of administration without compromising treatment efficacy or morbidity. Our meta-analysis of all published randomized trials found no difference in pain relief between single fraction and multifraction treatments [16]. Based on this information, the authors of this practice guideline conclude that a single fraction at 8 Gy is the preferred standard dose-fractionation for patients with uncomplicated painful bone metastases. In applying this evidence into practice, however, the following clinical factors merit consideration:

### 1. How durable is the pain relief?

Available evidence does not support the notion that a more durable response can be achieved with higher dose-fractionation [11].

### 2. Is the recommendation appropriate when preventing pathological response is an important consideration?

There is a lack of firm evidence relating the fractionation schedule to the prevention of pathologic fracture because no study evaluated the risk of pathologic fracture prior to treatment. Although the pathologic fracture rate was significantly higher after single fraction radiotherapy than after multifraction in the Dutch study [11], the absolute difference was only 2%. The RTOG study, on the other hand, showed a higher fracture rate following high dose-fractionation (40 Gy) than low-dose treatment (20 Gy) in patients with a solitary metastasis [22]. Until CT (computed tomography)-based bone density measurements [12] are correlated with pathologic fractures, no evidence-based recommendation can be given. Patients at risk of pathologic fractures in long or weight-bearing bones should be assessed by an orthopaedic surgeon. Where radiotherapy is considered for tumour downsizing prior to an orthopaedic procedure or for such patients who are not surgical candidates, fractionated treatment (e.g., 20 Gy/5 fractions, 30 Gy/10 fractions) would be considered appropriate by many clinicians. A discussion of fracture risk assessment is beyond the scope of this review but has been published elsewhere [23-25].

### 3. Does the recommendation apply to all pathologies?

It should be noted that the published studies included a heterogeneous group of patients differing in histologies, performance status, severity of pain, extent of disease, and so forth. The fact that breast, prostate, and lung cancer patients constituted the majority of trial patients implies a greater confidence in reproducing treatment results for these patients in practice. However, the evidence does not provide sufficient materials to allow a recommendation based on treatment outcomes among subgroups of different primary tumours or other patient- and tumour-related factors.

### 4. Do treatment field size and anatomical location affect application of the recommendation?

The evidence reviewed does not specifically address the results of large-volume (i.e. wide-field, hemibody irradiation), single fraction treatment. Although average treatment volumes were not reported in any of the single fraction trials, a significant proportion of patients did receive treatments to the lumbar spine and pelvis [10,11,13][Kirkbride P: Personal communications. 2001]. Since treatment volume was not an inclusion or exclusion criterion among those studies, it is reasonable to assume that study patients represent the majority of treatment volumes delivered in an average department. No difference in nausea and vomiting was seen in the subgroup of 133 patients from the Bone Pain Trial Working Party study, who were asked to self-assess nausea/vomiting experience in the first 14 days following treatments [10]. Therefore, the evidence does not support the choice of fractionated treatment based on volume consideration. However, the use of prophylactic ondansetron was shown to significantly reduce vomiting episodes in the single fraction arm compared with the 20 Gy arm (no prophylactic ondansetron) in the Canadian Bone Mets study [Kirkbride P: Personal communications. 2001]. For treatment over the epigastrium or lumbar spine, or with larger treatment volumes in the pelvis, it is reasonable to use a prophylactic antiemetic, as one would for hemibody irradiation. Patients may also be instructed to use anti-diarrheal agents if enteritis is experienced.

### 5. Do age and life expectancy affect application of the recommendation?

The underlying concern for this group of patients is whether single large-dose radiation compromises subsequent tolerance to re-irradiation. Although no untoward late effects were reported by the single fraction studies with follow-up of one year or more [10,11], clinicians may be uneasy about the long-term effects of repeated radiation. Given the lack of evidence to the contrary, single fraction radiotherapy remains an appropriate treatment option in this subgroup.

### 6. Does the presence of soft tissue disease around bone metastases affect application of the recommendation?

With CT/MRI (magnetic resonance imaging) diagnostic investigations becoming more routinely available, and the introduction of the CT-simulator into many departments, the extent of metastatic disease is likely to be better evaluated than in the past. In cases where lytic disease is associated with a large soft-tissue mass (e.g., in the acetabulum and adjacent pelvic bone), the desired palliative endpoint may be tumour shrinkage as well as pain control. No evidence-based recommendation can be given for this scenario.

### 7. When should re-irradiation be considered?

Re-irradiation may be considered in three scenarios: 1) no pain relief or pain progression after initial radiotherapy, 2) partial response with initial radiotherapy and the hope of achieving further pain reduction with more radiotherapy, and 3) partial or complete response with initial radiotherapy but subsequent recurrence of pain. The response after re-irradiation may be different for each of these scenarios. Two published studies reported response rates to re-irradiation [26,27] with doses ranging from 4 Gy as a single dose to 30 Gy in 10 fractions over two weeks. The Dutch Bone Metastases Study [11] was re-analyzed to separate the response to initial treatment from the response to re-treatment. Van der Linden presented the results at the 2003 ASTRO meeting [28]. The majority of the 173 patients re-irradiated received single fraction 8 Gy as the initial treatment. Overall response rate to re-treatment was 63%. At present no clear guideline can be given regarding dose-fractionation of re-irradiation. A new intergroup randomized trial supported by the National Cancer Institute of Canada (NCIC) of single versus multiple fractions for re-irradiation opened in January 2004 and is expected to accrue patients from Canada, the United Kingdom, the Netherlands, and Australia [29].

### 8. How is radiation-induced emesis best managed when delivering spinal irradiation?

No increase in acute gastrointestinal morbidity was observed with single fraction treatment compared to multiple fractions. The Canadian study showed significantly fewer vomiting episodes with single fraction treatment after prophylactic ondansetron was used in cases with treatment fields over the abdomen or pelvis [Kirkbride P: Personal communications. 2001]. Antiemetic agents should be considered as a prophylaxis, given that 30% or more of patients experienced vomiting following single or multifraction treatment in the two studies that specifically collected patient-assessed nausea/vomiting data [9,10].

## Conclusions

For patients where the treatment objective is pain relief, a single 8 Gy treatment, prescribed to the appropriate target volume, is recommended as the standard dose-fractionation schedule for the treatment of symptomatic and uncomplicated bone metastases. This recommendation applies to adult patients with single or multiple radiographically confirmed bone metastases of any histology corresponding to painful areas in previously non-irradiated areas without pathologic fractures or spinal cord/cauda equina compression. It does not apply to the management of malignant primary bone tumour. The following qualifying statements are provided to support the application of the recommendation in clinical practice:

• "*Standard*" refers to what is applicable to the majority of patients, with a preference for patient convenience and ease of administration and without compromising treatment efficacy or morbidity.

• The recommendation does not apply to lesions previously irradiated, or lesions causing cord compression or pathologic fractures, because such patients were mostly excluded from clinical trials examining fractionation schedules.

• Prophylactic antiemetic agents should be considered when a significant proportion of the gastrointestinal tract is in the irradiated volume.

• Patients and referring physicians should be advised that repeat irradiation to the treated area may be possible.

• There is insufficient evidence at this time to make a dose-fractionation recommendation for other treatment indications, such as long-term disease control for patients with solitary bone metastasis, prevention/treatment of cord compression, prevention/treatment of pathologic fractures, and treatment of soft tissue masses associated with bony disease.

This practice guideline incorporates recommendations based on a systematic review, comprehensive consideration of how the evidence may be applied to clinical practice, feedback from Ontario practitioners, and input from the Practice Guidelines Coordinating Committee prior to final approval. It is strongly endorsed by practitioners for whom it was developed.

## List of Abbreviations Used

ASTRO, American Society for Therapeutic Radiology and Oncology; CARO, Canadian Association of Radiation Oncologists; CT, computed tomography; Gy, gray(s); met, metastasis(es); MRI, magnetic resonance imaging; NCIC, National Cancer Institute of Canada; PGCC, Practice Guidelines Coordinating Committee; PGI, Practice Guidelines Initiative; RTOG, Radiation Therapy Oncology Group; SCGG, Supportive Care Guidelines Group.

## Competing interests

The authors declare that they have no competing interests.

## Authors' contributions

JW was the lead author responsible for designing and conducting the systematic review of the literature and the meta-analysis that informed the practice guideline, and for drafting and modifying the practice guideline report. JW was a member of the Supportive Care Guidelines Group and a Radiation Oncologist at the Juravinski Cancer Centre during the development of this guideline. RW reviewed all drafts of the guideline report and made major contributions to performing the meta-analysis that informed the practice guideline, and provided extensive input to the guideline as a radiation oncologist and methodologist. RW is co-Chair of the Supportive Care Guidelines Group. NL and MJ coordinated input from members of the SCGG, conducted literature searches, and drafted and edited the guideline report. MJ conducted duplicate data extraction and meta-analysis. NL updated the literature search, incorporated new data, conducted the practitioner feedback survey, and coordinated approval of the guideline by the Practice Guidelines Coordinating Committee. As members of the Supportive Care Guidelines Group, AB and TW provided substantial feedback on the guideline report at several points during its development, from both a radiation oncology and methodology perspective. Members of the SCGG provided feedback on all draft guideline reports.

## Pre-publication history

The pre-publication history for this paper can be accessed here:



## Figures and Tables

**Table 1:** Practitioner responses to eight items on the practitioner feedback survey.

**Item**	Number (% responders to survey*)		
	
	**Strongly agree or agree**	**Neither agree nor disagree**	**Strongly disagree or disagree**
The rationale for developing a clinical practice guideline, as stated in the "*Choice of Topic*" section of the report, is clear.	53 (98)	0	1 (2)
There is a need for a clinical practice guideline on this topic.	46 (85)	7 (13)	1 (2)
The literature search is relevant and complete.	51 (94)	3 (6)	0
The results of the trials described in the report are interpreted according to my understanding of the data.	44 (83)	5 (9)	4 (8)
The draft recommendations in this report are clear.	48 (91)	4 (8)	1 (2)
I agree with the draft recommendations as stated.	39 (74)	5 (9)	9 (17)
This report should be approved as a practice guideline.	39 (75)	5 (10)	8 (15)
If this report were to become a practice guideline, how likely would you be to make use of it in your own practice*?	**Very likely or likely**	**Unsure**	**Not at all likely or unlikely**
	42 (78)	6 (11)	6 (11)

* may not equal 100 percent due to rounding error
